# Genetic Architecture of Nitrogen-Deficiency Tolerance in Wheat Seedlings Based on a Nested Association Mapping (NAM) Population

**DOI:** 10.3389/fpls.2018.00845

**Published:** 2018-06-26

**Authors:** Deqiang Ren, Xiaojian Fang, Peng Jiang, Guangxu Zhang, Junmei Hu, Xiaoqian Wang, Qing Meng, Weian Cui, Shengjie Lan, Xin Ma, Hongwei Wang, Lingrang Kong

**Affiliations:** ^1^State Key Laboratory of Crop Biology, Shandong Key Laboratory of Crop Biology, College of Agronomy, Shandong Agricultural University, Tai’an, China; ^2^Lianyungang Academy of Agricultural Sciences, Lianyungang, China

**Keywords:** nitrogen utilization, nested association mapping (NAM), semi-wild wheat, quantitative trait loci (QTLs), wheat breeding

## Abstract

Genetic divergence for nitrogen utilization in germplasms is important in wheat breeding programs, especially for low nitrogen input management. In this study, a nested association mapping (NAM) population, derived from “Yanzhan 1” (a Chinese domesticated cultivar) crossed with “Hussar” (a British domesticated cultivar) and another three semi-wild wheat varieties, namely, “Cayazheda 29” (*Triticum aestivum* ssp. *tibetanum* Shao), “Yunnan” (*T. aestivum* ssp. *yunnanense* King), and “Yutian” (*T. aestivum petropavloski* Udats et Migusch), was used to detect quantitative trait loci (QTLs) for nitrogen utilization at the seedling stage. An integrated genetic map was constructed using 2,059 single nucleotide polymorphism (SNP) markers from a 90 K SNP chip, with a total coverage of 2,355.75 cM and an average marker spacing of 1.13 cM. A total of 67 QTLs for RDW (root dry weight), SDW (shoot dry weight), TDW (total dry weight), and RSDW (root to shoot ratio) were identified under normal nitrogen conditions (N^+^) and nitrogen deficient conditions (N^−^). Twenty-three of these QTLs were only detected under N^−^ conditions. Moreover, 23 favorable QTLs were identified in the domesticated cultivar Yanzhan 1, 15 of which were detected under N^+^ conditions, while only four were detected under N^−^ conditions. In contrast, the semi-wild cultivars contributed more favorable N^−^−specific QTLs (eight from Cayazheda 29; nine from Yunnan), which could be further explored for breeding cultivars adapted to nitrogen-deficient conditions. In particular, *QRSDW-5A.1* from YN should be further evaluated using high-resolution mapping.

## Introduction

Nitrogen (N), an essential plant nutrient, is vital for various aspects of crop growth and development, including seed germination, root architecture regulation, shoot development, flowering, and grain production (Lea and Morot-Gaudry, 2001; Alboresi et al., 2005; Kiba and Krapp, 2016; Yuan et al., 2016). Wheat production mainly depends on fertilizer input, particularly N fertilizer (Peng et al., 2009). From 2008 to 2015, the total global N consumption increased annually by 3.5%. In 2015, the total global N consumption was 223 million tons, and the average N application to wheat was 71–370 kg/hm^2^ (FAOSTAT, 2015), which is far higher than the safety threshold of 260 kg/hm^2^ (Liu, 2017) in many areas. This excessive N input not only raises the cost of production, but it also causes various soil and environmental issues (Peng et al., 2009). Thus, it is necessary that N use in agriculture is reduced without decreasing grain yields. Wheat varieties are typically developed for maximum production with high N fertilizer input, which results in a decrease in N use efficiency (Dong et al., 2014). Therefore, in order to increase production without further damage to the environment, high-yield crop varieties tolerant of N deficient conditions, or those that can efficiently utilize limited N, are desirable.

A thorough understanding of the mechanisms of N-deficiency tolerance and N-use efficiency in crop plants is required for the development of wheat varieties that are less dependent on N fertilizer. Plants have developed complex adaptive response pathways to cope with N fluctuations (Sandrine et al., 2014). N, as a metabolite, has been well studied. For instance, the nitrate transporter NRT1.1 not only transports NO_3_^−^, but also enhances the movement of basipetal auxin out of the roots, leading to the repression of lateral root development (Krouk et al., 2006; Wang et al., 2017). Many other proteins, such as the transcription factors ANR1 (nitrite regulator 1, Gan et al., 2005), SPL9 (squamosa promoter binding protein-like 9, Krouk et al., 2010), and NLP7 (NIN-like protein 7, Marchive et al., 2013), as well as the RING-type ubiquitin ligase NLA (Peng et al., 2007), respond to N metabolites. Transcriptome studies have shown that a wide range of physiological and developmental processes are controlled by N signals (Sandrine et al., 2014). Most N-responsive genes are also regulated by hormone and carbon signaling, indicating that N signaling mechanisms are highly integrated with other regulatory pathways (Sandrine et al., 2014). Despite this thorough understanding of N metabolites, the genetic mechanisms by which wheat tolerates or efficiently uses limited N are largely unclear.

Quantitative trait loci (QTL) mapping is a powerful tool for dissecting and understanding the genetic regulation of complex quantitative traits (Cui et al., 2014). Previous QTL studies have focused on morphological traits and crop yields in plants with low N tolerance or with efficient N uptake in hydroponic culture experiments (An et al., 2006; Laperche et al., 2007) and in field experiments (Quarrie et al., 2005; An et al., 2006; Laperche et al., 2007, 2008; Fontaine et al., 2009; Cui et al., 2014), leading to the identification of important QTLs on chromosomes 2A, 2B, 4A, 5A, 7A, and 7B. For instance, Quarrie et al. (2005) reported that major QTLs for grain yield components (ears per plant, grains per ear, and 1000s grain weight) under nitrogen deficiency condition were mapped on chromosomes 4AS, 7AL, 7BL, and around centromeres of chromosomes 4B and 6A using a spring wheat doubled haploid (DH) population derived from the cross Chinese Spring × SQ1. Laperche et al. (2007) detected 233 QTL for traits measured in each combination of environment and clustered into 82 genome regions, the dwarfing gene (*Rht-B1*), the photoperiod sensitivity gene (*Ppd-D1*) and the awns inhibitor gene (*B1*) coincided with regions that contained the highest numbers of QTL. Cui et al. (2016) reported that the *Rht-B1* affected not only plant height but also grain quality and its adaptability to N-deficient environments. Several other co-localizations between QTLs related to yield, physiological traits and enzyme activities involved in the control of N assimilation and recycling were detected for nitrate reductase (NR) and glutamate dehydrogenase (GDH) in maize (Hirel et al., 2001; Gallais and Hirel, 2004), glutamine synthetase (GS) in wheat (Habash et al., 2007; Fontaine et al., 2009). It is important to understand the identified specific QTLs associated with the adaptation of the plant to different N supply conditions. QTLs controlling high levels of N uptake and utilization can be detected specifically under high N conditions, and QTLs specifically detected under N limited conditions are involved in N-deficiency tolerance and adaption processes (Gaju et al., 2011). Direct selection for QTLs specifically detected under low N supply would be effective for the genetic improvement of N-deficiency tolerance traits (Dong et al., 2014).

Most studies were conducted on single, bi-parental population, thus the genetic polymorphisms are limited between two parents. Joint-multiple family analyses, such as “NAM,” potentially detect more QTLs, more accurately estimate QTL effects, better resolve QTL positions, and directly assess the distribution of functional allelic variation across multiple families, as compared to QTL analysis by bi-parental population (Yu et al., 2008; McMullen et al., 2009; Li et al., 2013; Ogut et al., 2015; Vatter et al., 2017). NAM population is a joint-multiple family comprising multiple bi-parental mapping families all sharing one common reference parent (Yu et al., 2008). For example, Sophie et al. (2015) developed a sorghum NAM population comprised of 2,400 recombinant inbred lines (RILs) from 10 families with the sorghum hybrid RTx430 as the common parent. The recombination rate of the NAM population was 4 cM/Mb, estimated based on 96,000 SNPs generated with a genotyping-by-sequencing approach, and 57,500 recombination events were observed (Sophie et al., 2015). Using this NAM population, Sophie et al. (2015) detected 41 QTLs and reduced the QTL mapping region to between 63 Kb and 1.9 Mb.

Here, we studied the genetic architecture of N-deficiency tolerance in wheat seedlings using a NAM dataset comprised of four related RIL populations. We constructed an integrative genetic map using high-density SNP markers genotyped with a 90K SNP chip. We aimed to detect QTLs involved in N-deficiency tolerance and to identify favorable alleles.

## Materials and Methods

### Plant Materials

The NAM population that we constucted was comprised of four RIL populations derived from crosses between a single female parent “Yanzhan 1” (YZ) and four different male parents. YZ is a good-quality, high-yield, disease-resistant variety of winter wheat cultivated in Henan Province of the Huanghuai region, China (in 2003). The male parents were “Hussar” (HR, a British dwarf cultivar), and three semi-wild wheat varieties from China: “Chayazheda” (CY, *Triticum aestivum* ssp. *tibetanum* Shao) from Tibet, “Yunnanxiaomai” (YN, *T. aestivum* ssp. *yunnanense* King) from Yunnan, and Yutiandaomai (YT, *T. aestivum petropavloski* Udats et Migusch) from Xinjiang. We crossed YZ with HR, CY, YN, and YT to develop separate RILs using a single seed descent approach. The final population sizes for each cross were 97, 82, 98, and 93, respectively.

### Experimental Design

All of the plants were grown in hydroponic culture (following Sun et al., 2013) in a greenhouse at Shandong Agricultural University, Shandong, China. We used Hoagland’s solution (Hoagland and Arnon, 1950) to optimize plant growth (**Supplementary Table S1**). To inhibit any potential nitrification of the nutrient solution, we added 2 mg/L dicyandiamide (a nitrification inhibitor). We tested two levels of N: normal (N^+^; 5.0 mmol/L N) and low (N^−^; 0.5 mmol/L N). We used a randomized complete block design with three replicates for each treatment. Wheat seeds were sterilized for 10 min in 10% sodium hypochlorite, washed with distilled water, and then germinated in a germination tray. After 7 days, one healthy seedling from each line and each treatment was transferred to a 200-cell bottomless tray. The tray was placed in an opaque plastic tank containing 20 L nutrient solution. The tank was opaque in order to encourage healthy root growth and to restrict the growth of algae. The nutrient solution was renewed every 3 days, and the pH was adjusted to 6.0 every day. We repeated this entire procedure six times in 2017: February 26–March 31 (E1); March 2–April 7 (E2); March 9–April 14 (E3); March 16–April 21 (E4); March 23–April 28 (E5); and March 31–May 5 (E6). Our experiment thus comprised 12 environmental combinations: E1N^+^, E1N^−^, E2N^+^, E2N^−^, E3N^+^, E3N^−^, E4N^+^, E4N^−^, E5N^+^, E5N^−^, E6N^+^, and E6N^−^.

### Trait Measurements

All of the plants were harvested after 30 days in the nutrient solution. The roots were cleaned with distilled water, and excess water was blotted with absorbent paper. Plants were then dried for 24 h at 56°C in a drying oven before measuring dry root weight (RDW, in mg) and dry shoot weight (SDW, in mg). The total dry weight (TDW, in mg) was calculated as RDW + SDW and the ratio of dry root weight to dry shoot weight (RSDW) was calculated as RDW/SDW. To estimate the plant response to N deficiency, we calculated a “global” interaction variable (G × N) as (N_y_^−^−N_y_^+^)/N_y_^+^, where N_y_^−^ and N_y_^+^ represent the trait values in the N^−^ and N^+^ treatments, respectively.

### Genotyping and Genetic Map Construction

Genomic DNA was extracted from the seedling leaves of all five parents and all of the RILs (Doyle and Doyle, 1987). DNA samples were genotyped with an Illumina 90K assay (Wang et al., 2014). All of the SNP markers for each line were converted based on the alleles of the parents: ‘A’ for the common parent YZ, ‘B’ for the other parental lines, ‘H’ for the heterozygous genotype, and ‘-’ for missing information. Individual genetic maps for each RIL population were constructed using Kosambi mapping (Kosambi, 1944) and individual maps were combined with Joinmap v4.0 (Van Ooijen and Jansen, 2013)^1^. The integrative map was drawn using MapChart v2.2 (Voorrips, 2002)^2^.

### Data Analysis and QTL Mapping

We tested the significance of the phenotypic differences between each pair of parents using Student’s *t-*tests. To estimate the variance across genotypes (G), environment (E), genotype/environment interactions (GEI), and replicates, we used analysis of variance (ANOVA) with generalized linear models (GLMs) in SPSS v20 (Bryman and Cramer, 2012)^3^. Heritability (h^2^) was computed using the estimated variance components V_G_ /(V_G_ + V_GEI_/s + V_e_/sr), where V_G_, V_GEI_, and V_e_ are the variances of G, GEI, and the residuals, respectively; s is the number of environments; and r is the number of replicates. The best linear unbiased estimates (BLUE) for each line with respect to each trait across all traits were used to analyze pairwise correlations. We mapped QTLs with the ICIM-ADD method (Li et al., 2007) using stepwise regression, and considered all of the marker information simultaneously in Ici-Mapping v4.1 (Li et al., 2007)^4^. We used a walking speed of 1.0 cM for all of the QTL calculations, and a stepwise regression probability (*P*-value inclusion threshold) of 0.001. We considered a QTL to be present if the limit of detection (LOD) was >2.5 in the NAM population, and >2.0 in at least one RIL population.

## Results

### Phenotypic Variation

**FIGURE 1:**
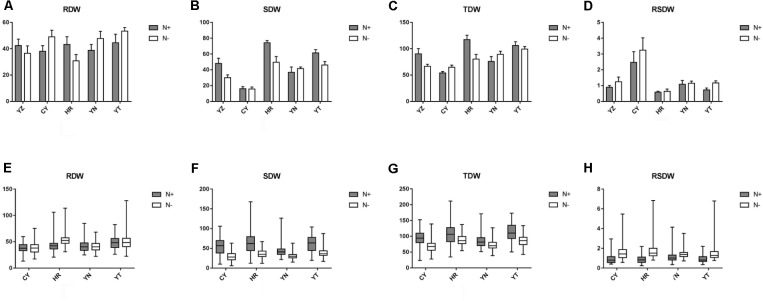
Performance of the five parental varieties and the phenotypic variations of each RIL population with respect to the measured seedling growth traits. **(A–D)** The performance of the five parental varieties is shown using a histogram. **(E–H)** The phenotypic variation of the RIL populations is shown using a boxplot. RDW indicates root dry weight; SDW indicates shoot dry weight; TDW indicates total dry weight; RSDW indicates root to shoot ratio. Performance under N^+^ (normal N supply) and N^−^ (low N supply) conditions are shown in a gray and white pattern. The *x*-axis represents five parental lines: YZ (Yanzhan 1), CY (Cayazheda), HR (Hussar), YN (Yunnanxiaomai), and YT (Yutiandaomai).

The traits of the five parents differed substantially under both N^+^ and N^−^ conditions across all of the treatments and environments, and exhibited distinctly different responses to N deficiency (**Figures 1A–D** and **Supplementary Table S2**). For instance, under N^−^ conditions, both the RDW and SDW of the common parent YZ were reduced, but RDW increased in YT and SDW increased in YN, suggesting that these parental species possessed different levels of N-deficiency tolerance (**Figures 1A–D** and **Supplementary Table S2**). Strong transgressive segregation was observed in all of the RIL populations, indicating that favorable alleles were distributed among the parents. Considerable continuous variation was observed in all of the measured traits across all of the populations (**Figures 1E–H** and **Supplementary Tables S2**, **S3**). For each parent and RIL population, the differences in SDW between the N^+^ and N^−^ conditions were significantly greater than the differences in RDW (one-way ANOVA, *P* < 0.001, **Figure 1**; **Supplementary Table S3**). The estimated *h^2^* of all of the traits ranged from 18.5% for RSDW to 74.0% for TDW. The *h^2^* of TDW, RDW, and SDW was high (mostly > 40%) under both N^+^ and N^−^ conditions. The *h^2^* of RSDW was lower, however, ranging from 18.5 to 47.8%. For each trait, *h^2^* varied between populations. Our results suggested that all of the measured traits were affected not only by genotype, environment, and GEI, but also by genetic background (**Supplementary Table S3**). The phenotypic pairwise correlations between the measured traits were similar under both N^+^ and N^−^. RDW and SDW were positively correlated with each other and with TDW. RSDW was positively correlated with RDW (as expected), but negatively correlated with SDW (**Table 1**).

**Table 1:** Pairwise phenotypic correlations among the measured seedling traits.

	RDW-N^+^	RSDW-N^+^	SDW-N^+^	TDW-N^+^	RDW-N^−^	RSDW-N^−^	SDW-N^−^	TDW-N^−^
RDW-N^+^		0.184^**^	0.296^**^	0.651^**^	0.282^**^	0.038	0.226^**^	0.309^**^
RSDW-N^+^			−0.686^**^	−0.470^**^	−0.083	0.250^**^	−0.329^**^	−0.243^**^
SDW-N^+^				0.918^**^	0.318^**^	−0.221^**^	0.563^**^	0.528^**^
TDW-N^+^					0.366^**^	−0.156^**^	0.533^**^	0.542^**^
RDW-N^−^						0.440^**^	0.376^**^	0.844^**^
RSDW-N^−^							−0.518^**^	−0.028
SDW-N^−^								0.810^**^

*^∗∗^Indicates significance at P ≤ 0.01.*

### The Novel Genetic Map

**FIGURE 2:**
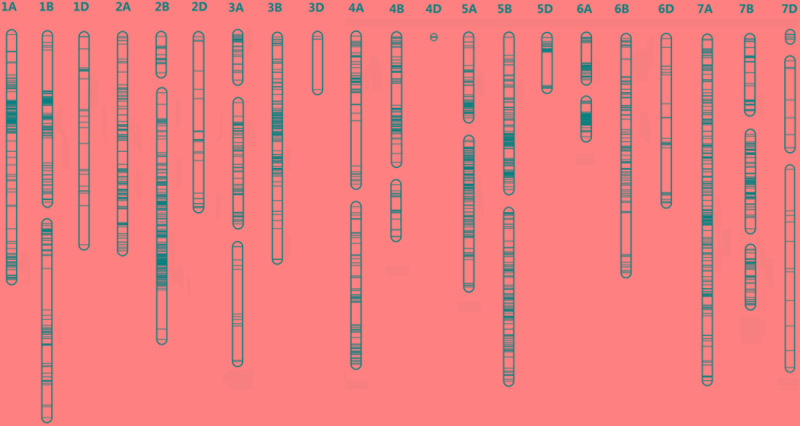
The integrated genetic linkage map of the NAM population. The integrated genetic map of wheat was developed using a NAM population derived from the cross of Yanzhan 1 with four other parents. The positions of the marker loci are indicated using black lines on the chromosome.

We selected several polymorphic markers distributed across all 21 chromosomes for linkage analysis: 548 for CY, 1,127 for YN, 1,514 for YT, and 1,595 for HR. We mapped 2,059 loci, including 34 linkage groups, to our integrated genetic map (**Figure 2**, **Table 2**, and **Supplementary Table S4**), with a total coverage of 2,355.75 cM and an average marker spacing of 1.13 cM. Our integrated map included three genomes: the A genome was 887.67 cM (38.0%), and contained 946 loci (45.89%); the B genome was 955.34 cM (40.90%), and contained 979 loci (47.55%); and the D genome was 492.74 cM (21.1%), and contained 135 loci (6.06%). The chromosome sizes ranged from 0.61 cM (chromosome 4D) to 186.86 cM (chromosome 1B). Chromosome 2B had the most loci (202), while chromosome 3D had the least (4). We obtained good coverage for the A and B genomes, but few polymorphic loci were identified for the D genome. Our integrated linkage map had greater genome coverage, more markers, and lower average maker distance than the individual maps (**Table 2**).

**Table 2:** The novel integrated genetic linkage map of the NAM population.

Chromosome	CY			HR			YN			YT			Integrated map		
	Coverage (cM)	Markers No.	Average spacing (cM)	Coverage (cM)	Markers No.	Average spacing (cM)	Coverage (cM)	Markers No.	Average spacing (cM)	Coverage (cM)	Markers No.	Average spacing (cM)	Coverage (cM)	Markers No.	Average spacing (cM)
1A	65.7	22	2.99	91.77	31	2.96	77.8	29	2.68	103.24	105	0.98	126.41	145	0.87
1B	52.54	25	2.1	64.99	25	2.6	95.52	54	1.77	78.51	46	1.71	186.86	139	1.34
1D	38.93	4	9.73	81.28	18	4.52	12.34	6	2.06	8.81	6	1.47	107.51	24	4.48
2A	87.5	24	3.65	135.49	48	2.82	78.6	32	2.46	70.76	30	2.36	110.61	96	1.15
2B	6.54	5	1.31	92.17	77	1.2	132.27	72	1.84	77.51	84	0.92	147.90	212	0.70
2D	3.51	2	1.75	53.48	11	4.86	11.08	5	2.22	38.67	13	2.97	86.92	27	3.22
3A	30.12	13	2.32	69.6	45	1.55	158.83	51	3.11	19.43	16	1.21	150.92	96	1.57
3B	163.64	39	4.2	76.43	32	2.39	91.67	53	1.73	49.97	6	8.33	115.48	107	1.08
3D	–	–	–	28.73	3	9.58	1.68	3	0.56	–	–	–	30.32	4	7.58
4A	6.73	3	2.24	86.63	38	2.28	98.37	35	2.81	76.52	45	1.7	158.20	107	1.48
4B	0.65	2	0.32	90.37	53	1.71	78.52	35	2.24	19.9	16	1.24	95.38	89	1.07
4D	–	–	–	0.6	5	0.12	0.62	2	0.31	–	–	–	0.61	5	0.12
5A	112.87	39	2.89	108.23	57	1.9	93.64	47	1.99	65.89	80	0.82	121.59	186	0.65
5B	57.73	8	7.22	130.48	48	2.72	136.46	80	1.71	189.31	104	1.82	167.19	212	0.79
5D	4.7	6	0.78	28.41	15	1.89	5.78	6	0.96	1.62	9	0.18	28.58	25	1.14
6A	36.21	10	3.62	9.83	29	0.34	15.68	21	0.75	41.4	68	0.61	45.13	113	0.40
6B	8.51	6	1.42	103.48	28	3.7	99	37	2.68	58.97	27	2.18	121.05	86	1.41
6D	29.8	9	3.31	21.23	5	4.25	44.55	8	5.57	41.3	9	4.59	85.35	28	3.05
7A	61.87	10	6.19	141.9	80	1.77	202.47	68	2.98	128.69	82	1.57	174.82	202	0.87
7B	22.5	15	1.5	118.61	67	1.77	74.79	33	2.27	55.25	47	1.18	121.47	134	0.91
7D	5.81	4	1.45	114.25	15	7.62	47.04	7	6.72	4.58	29	0.16	153.45	22	6.98
Total	795.85	246	3.24	1647.92	730	2.26	1556.74	684	2.28	1130.31	822	1.38	2355.75	2059	1.13

### QTL Mapping for Seedling Growth Traits

**FIGURE 3:**
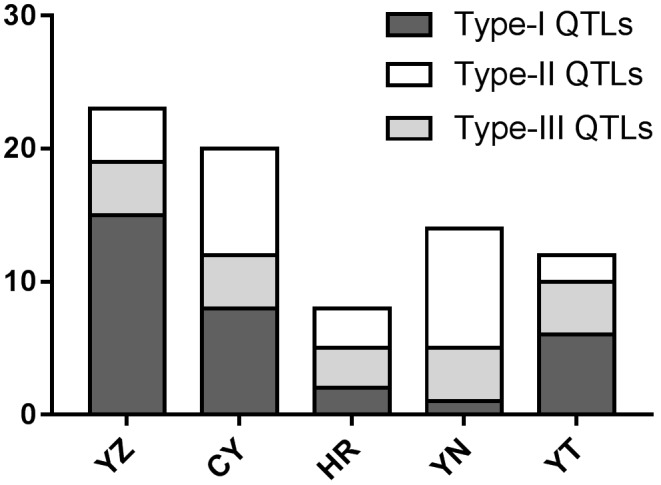
Statistics of favorable QTLs/allele donated by the parents in this study. The identified QTLs were divided into three types: Type-I (QTLs detected only under N^+^ conditions), Type-II (QTLs detected only under N^−^ conditions), and Type-III (QTLs detected under both conditions). Statistics of the Type-I, Type-II, and Type-III QTLs are indicated with gray, white, and light gray columns, respectively. The *x*-axis represents the five parental lines: YZ (Yanzhan 1), CY (Cayazheda), HR (Hussar), YN (Yunnanxiaomai), and YT (Yutiandaomai).

We detected 67 QTLs affecting seedling growth traits, including 31 QTLs identified only under N^+^ treatment, 22 only under N^−^ treatment, and 14 detected under both (**Table 3**, **Supplementary Tables S5**, **S6**, and **Supplementary Figure S1**). The 67 QTLs were distributed across all 21 chromosomes, except 2D, 3D, 4B, 4D, 5D, and 7D. The phenotypic variance (PVE) explained by these QTLs ranged from 2.3% (SDW in E5N^+^) to 38.0% (SDW in E3N^+^). The 44 QTLs with PVEs greater than 10% (identified as “primary QTLs”) were mainly concentrated on chromosomes 1B, 2A, 2B, and 3A. Statistics of the favorable QTLs donated by parents are shown in **Figure 3** and **Supplementary Table S4**. Twenty-three favorable QTLs were donated by the domesticated cultivar of YZ, in which 15 were detected only under N^+^ conditions, and four were detected only under N^−^ conditions. The semi-wild cultivars CY and YN contributed eight and nine favorable QTLs detected only under N^−^ conditions.

**Table 3:** Stable additive QTLs for seedling traits in the NAM population.

Chromosome	Position	Right maker	Left maker	QTL detected for N^+^, N^−^ treatments			Comparison with previous studies	
				N^+^	N^−^	N^+^ and N^−^	QTL	References
1A	115	*BobWhite_c12305_959*	*wsnp_Ex_c11939_19147790*		***QRDW-1A.1*** ***QTDW-1A.1***		*QTdw-1A.1*	Sun et al., 2013
							*QTdw.1*, *QRdw.1*	Guo et al., 2012
1A	120	*BS00062759_51*	*BS00063847_51*			***QRSDW-1A.1***		
1B	37	*Tdurum_contig20299_368*	*BobWhite_c48550_198*		*QRSDW-1B.1* ***QSDW-1B.1***			
1B	42	*wsnp_Ex_c5098_9047611*	*BS00063092_51*		*QRSDW-1B.2*		*SDW-H*, *NUP-H, RDW-H*,	An et al., 2006
1B	44	*tplb0048b10_1365*	*Ku_c28580_432*	*QRDW-1B.1*	*QRSDW-1B.3*			
1B	50	*RAC875_c1785_366*	*BS00082071_51*	***QSDW-1B.2***				
1B	52	*wsnp_Ex_c1440_2764269*	*BS00072289_51*	***QRDW-1B.2***				
1B	58	*wsnp_Ex_c21559_30710510*	*Kukri_c44191_452*			***QSDW-1B.3***	*TN-H*	An et al., 2006
							*cTRL*	Laperche et al., 2006
1B	1	*Kukri_c8390_1102*	*BS00080212_51*			***QSDW-1B.4***		
1B	55	*Ex_c29452_302*	*BobWhite_c20073_382*			*QRSDW-1B.4*		
1D	26	*BS00042197_51*	*RAC875_c62_1514*		***QRDW-1D.1***		*Qsnp-1D*	Xu et al., 2014
							*RFW, RDW*	Zhang et al., 2013
2A	31	*IAAV7468*	*Tdurum_contig66015_346*		*QRDW-2A.1*		*R-GS*	Fontaine et al., 2009
							*QTkw-2A.1, QKl-2A.1*	Cui et al., 2014
2A	48	*RAC875_c20700_853*	*RAC875_c13116_943*	***QTDW-2A.1***		*QSDW-2A.1*	*QTkw-2A.2*	Cui et al., 2014
2A	52	*Tdurum_contig47258_1039*	*wsnp_Ex_c22645_31845564*	***QRDW-2A.2***	*QSDW-2A.2* *QTDW-2A.2*		*QKnps-2A.2*	Cui et al., 2014
2A	72	*Ra_c26702_797*	*Excalibur_c96_619*	***QRDW-2A.3***			*NS%, GPC*	Laperche et al., 2007
2A	75	*BobWhite_c16923_64*	*RAC875_c104160_61*			***QRDW-2A.4***		
2A	76	*Tdurum_contig60205_806*	*Excalibur_rep_c66618_87*	***QSDW-2A.3***				
2A	81	*Ku_c13700_1196*	*BS00107804_51*	*QRDW-2A.5*		***QTDW-2A.3***		
2A	84	*IAAV2861*	*wsnp_Ex_rep_c70299_69243401*	***QRDW-2A.6***	*QTDW-2A.4*		*SDW*	Zhang et al., 2013
2A	94	*BobWhite_c17783_174*	*CAP7_c1527_136*	***QRDW-2A.7***				
2A	97	*Tdurum_contig47508_250*	*BS00053834_51*	***QRDW-2A.8***				
2B	10	*BS00099658_51*	*Excalibur_c17250_592*	***QSDW-2B.1***			*QGY-2B*	Xu et al., 2014
							*R-GS, A-GS, A-DTH*	Fontaine et al., 2009
							*LA, cTDM, tADM*	Laperche et al., 2006
2B	40	*Ex_c12051_875*	*BS00041585_51*		*QSDW-2B.2*			
2B	73	*RFL_Contig4849_702*	*BS00066545_51*	***QRDW-2B.1***				
								
2B	77	*RFL_Contig1987_3440*	*Tdurum_contig26542_457*	***QTDW-2B.1***				
2B	78	*Excalibur_rep_c66577_159*	*RFL_Contig4718_1269*	***QRDW-2B.2***				
2B	80	*GENE-4029_80*	*wsnp_BE490763B_Ta_2_1*			***QRDW-2B.3***	*R-GDH, R-NH4*	Fontaine et al., 2009
							*TDM, tRDM, TRM, TRL*, *RUE*	Laperche et al., 2006
2B	91	*Tdurum_contig28795_322*	*wsnp_Ex_c45468_51254832*		***QTDW-2B.2***			
2B	93	*RAC875_c35778_201*	*BS00094578_51*		***QTDW-2B.3***			
2B	94	*BS00022374_51*	*BS00009060_51*	***QRDW-2B.4***				
2B	98	*Excalibur_c42146_266*	*BS00026432_51*		***QTDW-2B.4***			
2B	108	*BS00060618_51*	*wsnp_RFL_Contig2324_1803878*	***QRDW-2B.5***				
3A	4	*Excalibur_c34889_526*	*Jagger_c6722_104*	***QRDW-3A.1***				
3A	7	*RAC875_c787_431*	*Ku_c17560_91*	***QRDW-3A.2***				
3A	10	*BS00036492_51*	*BS00022129_51*		*QRSDW-3A.1*	***QSDW-3A.1*** ***QRDW-3A.3 QTDW-3A.1***	*TN-H, RDW-H*	An et al., 2006
							*GPA-3A*	Laperche et al., 2007
3B	63	*wsnp_Ku_c29102_39008953*	*wsnp_Ex_c64005_62986957*	*QTDW-3B.1*				
3B	115	*IAAV8892*	*BS00071041_51*	***QRDW-3B.1***			*QTKW.3B*	Xu et al., 2014
							*QRFW.WL.3B*	Zhang et al., 2013
4A	17	*BobWhite_rep_c66057_98*	*wsnp_Ex_c5690_9994305*			***QRDW-4A.1***	*NUP-LN2*	An et al., 2006
4A	21	*Ra_c7973_1185*	*IAAV4609*	*QTDW-4A.1*				
4A	22	*Ku_c11865_406*	*wsnp_Ex_c31508_40288653*	***QSDW-4A.1***				
5A	24	*BS00047242_51*	*Excalibur_rep_c95828_165*	*QTDW-5A.1*				
5A	30	*wsnp_Ex_c18941_27840714*	*Tdurum_contig10601_289*		*QRSDW-5A.1*		*NUP-HN1*	An et al., 2006
5B	30	*RAC875_c29907_115*	*BS00015136_51*	***QTDW-5B.1***				
6B	10	*wsnp_Ku_c24391_34351602*	*Kukri_c23491_274*	*QSDW-6B.1*			*NUP-LN1*	An et al., 2006
6B	19	*CAP11_c7959_386*	*wsnp_Ex_c7191_12352173*	***QRSDW-6B.1***	*QRDW-6B.1*			
6B	48	*wsnp_Ra_c20409_29673950*	*BS00033629_51*	***QRSDW-6B.2***				
6D	85	*BS00022787_51*	*IACX5958*	***QTDW-6D.1***				
7A	7	*Tdurum_contig5646_929*	*Excalibur_c20311_240*		*QRDW-7A.1*		*NS%-7A1*	Laperche et al., 2007
							*SFW*	Zhang et al., 2013
							*QTkw-7A.2*	Cui et al., 2014
7A	90	*RFL_Contig5285_365*	*BS00108184_51*			***QTDW-7A.1***	*QSLFW.WY.7A*	Zhang et al., 2013
7A	105	*Excalibur_c47990_159*	*wsnp_Ku_c10202_16937059*	***QRDW-7A.2***			*NUP-H*	An et al., 2006
7A	113	*CAP7_c7296_88*	*BobWhite_c5125_258*			*QTDW-7A.2*		
7B	0	*wsnp_Ex_c7934_13467460*	*Tdurum_contig55961_526*		*QSDW-7B.2* *QTDW-7B.1*		*NUR*	Laperche et al., 2006
							*NUP*	Laperche et al., 2006
7B	35	*BobWhite_c10448_80*	*Jagger_c9314_100*	***QSDW-7B.1***			*NS%-7B1*	Laperche et al., 2007
							*cLRN_PRL*	Laperche et al., 2006

^a^*QTLs marked with a bold typeface represent primary QTLs.*

In multiple environments, we repeatedly detected 18 QTLs for TDW on chromosomes 1A, 2A, 2B, 3A, 3B, 4A, 5A, 5B, 6D, 7A, and 7B. These 18 QTLs included eight identified only under the N^+^ treatment, seven only under the N^−^ treatment, and three under both treatments. Most of the favorable alleles (those that increased the value of a given trait) were donated by parent CY. Four QTLs (*QTDW-2A.3*, *QTDW-3A.1 QTDW-7A.1*, and *QTDW-7B.1*) were regarded as primary QTLs, as they explained 10.5–31.7% of the phenotypic variation.

We identified 26 QTLs for RDW on chromosomes 1A, 1B, 1D, 2A, 2B, 3A, 3B, 4A, 6A, 6B, and 7A. Of these 26, 16 were only detected under N^+^ treatment, six were only detected under N^−^ treatment, and four were detected under both N^+^ and N^−^ treatments. Most of these QTLs explained more than 10% of the phenotypic variation. Two QTLs detected in the HR population, *QRDW-2A.2* and *QRDW-7A.2*, accounted for 18.0–34.6% and 23.2–31.9% of the phenotype variance, respectively. Most of the favorable alleles were donated by parent YZ.

The QTLs for SDW were detected on chromosomes 1B, 2A, 2B, 3A, 4A, 6B, and 7B. These QTLs included six under N^+^ treatment only, four under N^−^ treatment only, and four under both treatments. The favorable alleles were mainly donated by parent YZ. We identified four primary QTLs: *QSDW-2A.1*, *QSDW-2A.3*, *QSDW-2B.1*, and *QSDW-3A.1*.

We identified nine QTLs for RSDW on chromosomes 1A, 1B, 3A, 5A, and 6B. These included two QTLs under N^+^ treatment only, five under N^−^ treatment only, and two under both treatments. Four primary QTLs (*QRSDW-1A.1*, *QRSDW-1B.1*, *QRSDW-6B.1*, *QRSDW-6B.2*) were identified with PVEs ranging from 10.3 to 31.0%.

## Discussion

### NAM Population and the Novel Integrated Genetic Map

The five parents of the NAM population in this study constitute local adaptable varieties of different origins, and exhibit high phenotypic and genetic diversity. The common parent of YZ was domesticated with the features of a short lifecycle and high yield (Yao et al., 2010). HR (Squadron/Rendezvous) is a British dwarf cultivar developed by Cambridge Plant Breeders (Cambridge, United Kingdom) and Syngenta (formerly Imperial Chemical Industries) that is resistant to many wheat diseases, but was domesticated with a longer lifecycle (Wilde et al., 2008). The other three semi-wild parents are wheat germplasm resources unique to Western China, and possess many morphological characteristics that differ significantly from common wheat, such as late-flowering, brittle rachis when naturally mature, hard glumes, high protein content, and barren tolerance (Sun et al., 1998; Chen et al., 2007; Zeng et al., 2010; Guo and Han, 2014). The use of this NAM population increased the number of QTLs identified and enhanced the mapping resolution in comparison to the bi-parental population analyses. We constructed an integrated map of the NAM population using 2,059 SNP markers with an average marker spacing distance of 1.13 cM. The novel integrated genetic map shows good genome coverage, high density, and good collinearity with physical maps, and is thus more suitable for genetic research than the four individual genetic maps.

### Mapping of QTLs Involved in N Deficiency Tolerance

N uptake and utilization at the seedling stage are important for accumulating a N reservoir, which then fulfills the N requirements during plant growth until the maturity stage (Lian et al., 2005). Genotype selection based on comprehensive performance under N^+^ and N^−^ conditions would be valuable for evaluating N deficiency tolerance (Fontaine et al., 2009; Wang et al., 2017). In this study, we tested two different N supply levels, namely, N^+^ (normal nitrogen supplement) and N^−^ (low nitrogen supplement), under hydroponic culture conditions. The identified QTLs could be divided into three types: Type-I (QTLs detected only under N^+^ conditions), Type-II (QTLs detected only under N^−^ conditions), and Type-III (QTLs detected under both conditions). We identified 14 Type-III QTLs that were indispensable for constitutive processes, with polymorphisms existing between their parents (Laperche et al., 2007). Thirty-one Type-I QTLs identified in our study were assumed to be associated with high levels of N uptake or utilization. Fifteen favorable alleles of Type-I QTLs were donated by parent YZ. *QRDW-2A.2* was mapped to the chromosomal region associated with NS% (straw nitrogen content) and GPC (grain protein content) reported by Laperche et al. (2007). *QSDW-6B.1* and *QRDW-7A.2* have been reported to affect NUP (root N content; An et al., 2006). We detected 23 Type-II QTLs involved in N-deficiency tolerance. These favorable alleles were mainly donated by parents CY and YN. Type-II QTLs, namely *QRSDW-1B.2*, *QRDW-1D.1*, *QRDW-2A.1*, *QSDW-2A.2*, *QTDW-2A.2*, *QRSDW-5A.1*, *QSDW-7B.2*, and *TDW-7B.1*, have been reported to influence N deficiency tolerance and related traits in previous studies (An et al., 2006; Laperche et al., 2006, 2007; Habash et al., 2007; Fontaine et al., 2009; Guo et al., 2012; Sun et al., 2013; Zhang et al., 2013; Cui et al., 2014; Xu et al., 2014). The remaining common QTLs/genome regions are listed in **Table 3**. The coincidence of the QTLs across different genetic backgrounds not only implies the reliability of the QTLs detected in this study, but also highlights the importance of the chromosomal region.

The “global” interaction variable has previously been used to characterize plant responses to stress (Yan et al., 1999; Yadav et al., 2003; Lian et al., 2005; Laperche et al., 2006, 2007). In this study, we compared two QTL sets detected under the two N levels, from which 23 Type-II QTLs were discovered to be involved in N deficiency tolerance. To further distinguish the QTLs specifically involved in the adaptation of wheat to N deficiency, the “global” interaction variable of (N_y_^−^−N_y_^+,^)/N_y_^+^ was alternatively used for QTL detection. We hypothesized that the QTLs identified both by the “global” interaction variable and by the N^−^ treatments constituted high confidence QTLs involved in N deficiency tolerance. Four QTLs (*QRSDW-1B.2*, *QRDW-2A.1*, *QRSDW-5A.1*, and *QRDW-7A.1*) were identified that met these criteria, and have previously been reported to influence several traits (**Table 3**), including SDW, NUP (root N content), RDW (An et al., 2006), NUP, and NS% (Laperche et al., 2007), R-GS (glutamine synthetize activity, Fontaine et al., 2009), and TKW (thousand kernel weight, Cui et al., 2014).

### Implications for Breeding

The size and topology of the root system determines the N uptake ability of the plant (Lea and Morot-Gaudry, 2001). When N is limited or deficient, wheat responds by increasing root growth and proliferation at the expense of the shoots, leading to high root/shoot ratios (Ericsson, 1995; Ameziane et al., 1997). As N uptake during the vegetative stage plays an important role in plant growth even into maturity, breeders select wheat genotypes that perform well under both N^+^ and N^−^ conditions (Fontaine et al., 2009). In the NAM population, the phenotypic variation of the parents resulted in a rich allelic variation in response to N fluctuation. RDW, SDW, and TDW were high in the parents YZ, HR, and YT under N^+^ conditions, but these traits decreased substantially under N^−^ conditions. In comparison, RDW, SDW, and TDW were lower in parents CY and YN under N^+^ conditions, but increased under N^−^ conditions. Many RILs had greater RDW, SDW, and TDW under N^−^ conditions than under N^+^ conditions; for instance, RDW, SDW, and TDW were greater in many RILs under N^−^ conditions in comparison to their parents. This can be explained as the pyramiding of favorable alleles from both parents, which is valuable for the breeding of wheat varieties tolerant of low N levels.

To develop wheat varieties adapted to limited or deficient N conditions, direct selection for favorable QTLs specifically detected under N^−^ condition is effective. We identified eight primary QTLs (*QRDW-1A.1*, *QTDW-1A.1*, *QSDW-1B.1*, *QRDW-1D.1*, *QTDW-2B.2*, *QTDW-2B.3*, *QTDW-2B.4*, and *QRDW-6A.1*), all of which are probably involved in N-deficiency tolerance. These QTLs are of value in wheat breeding programs designed to increase N deficiency tolerance. Moreover, N uptake or utilization traits have been considered as indirect selection criteria for the improvement of N-deficiency tolerance (Lian et al., 2005; Fontaine et al., 2009; Wang et al., 2017). In this study, we also identified 25 primary QTLs implicated in N uptake and utilization, and 11 primary QTLs associated with constitutive process (**Table 3**). For instance, *QTDW-3A.1* showed multiple effects on biomass, grain number and yield in the mature periods; *QTDW-5A.1* was also mapped to chromosomal region affecting thousand kernel weight in the mature periods (**Supplementary Table S7**). The QTLs also could be used in breeding programs by pyramiding the different types of QTLs or by using pleiotropic QTLs through MAS. Thus, the mapped QTL interval markers could be used in MAS after being converted into high-throughput KASP (Kompetitive Allele Specific PCR) markers.

In this study, 23 favorable QTLs were donated by the domesticated cultivar of Yanzhan 1, in which 15 were Type-I (detected only in N^+^ conditions) and only four were Type-II (detected only in N^−^ conditions). In contrast, the semi-wild cultivars contributed more favorable Type-II QTLs, including eight favorable QTLs from CY and nine from YN. Seven Type-II favorable QTLs (*QRSDW-1B.1*, *QSDW-1B.1*, *QRSDW-1B.3*, *QTDW-2B.2*, *QTDW-2B.3*, *QTDW-2B.4*, and *QRDW-6B.1*), donated by CY and YN, are novel QTLs that have not been reported in previous studies. The modern variety (YZ) possessed more favorable QTLs/genes for N uptake and utilization under N^+^ conditions, while the semi-wild wheat varieties were more likely to have favorable QTLs/genes for N-deficiency tolerance (**Figure 3** and **Supplementary Table S8**). This indicates that a domesticated selection might have occurred in the breeding process. Modern domesticated varieties are supplied with adequate N during yield experiments, and therefore lines that use more N to increase yield are more likely to be selected for cultivation. The semi-wild wheat varieties were from wilderness areas with limited N, and are thus subject to strong evolutionary pressure to maintain N-deficiency tolerance. Semi-wild wheat varieties are therefore an important genetic resource that can be used to improve the N-deficiency tolerance of modern varieties.

The “global” interaction variable identified *QRSDW-1B.2*, *QRSDW-5A.1*, and *QRDW-7A.1* as high confidence QTLs involved in N stress adaption, with favorable alleles donated by the semi-wild wheat YN. *QRSDW-5A.1* with positive alleles increased the RSDW value from 18.9% to 22.7%, indicating tremendous potential for its application in wheat breeding programs designed to increase N-deficiency tolerance. We predicted that the candidate genes for *QRSDW-5A.1* might be within the 0.7 cM confidence interval of *wsnp_Ex_c18941_27840714*–*Tdurum_contig10601_289*. Based on our integrated genetic map, which had high density and good collinearity with the physical map, we further compared the overlapping intervals of the collocated QTL peaks with the IWGSC RefSeq Annotations database v 1.0.^5^ The confidence intervals of *wsnp_Ex_c18941_27840714*–*Tdurum_contig10601_289* spanned 0.8 Mb (5A: 547647367–548503773). This region harbors 12 annotated genes in wheat (**Supplementary Table S9**), most notably an auxin responsive gene (*ARF*) cluster (including eight genes), which might be a candidate for *QRSDW-5A.1.* This information provides a reference for the future high-resolution mapping and map-based cloning of *QRSDW-5A.1*.

## Conclusion

A NAM population comprised of four RIL populations was used for QTL mapping. An integrated genetic map of wheat, with high density and good collinearity with the physical maps, was developed. The NAM population was highly variable for all of the measured traits. Many RILs tolerant of N deficiency exhibited high RDW, SDW, and TDW under the N^−^ treatment. We detected 31 QTLs under N^+^ conditions that are possibly involved in N uptake or utilization, with favorable alleles mainly donated by the modern parent YZ. We detected 23 QTLs under N^−^ conditions, possibly associated with N-deficiency tolerance, with most of the favorable being alleles donated by the semi-wild parents CY and YN. Four QTLs detected under N^−^ conditions were identified as high confidence QTLs involved in N-deficiency tolerance. A domesticated selection might have occurred during the breeding process. Semi-wild wheat varieties constitute an important genetic resource that can be used to improve the N-deficiency tolerance of modern varieties.

## Author Contributions

DR and XF designed the experiments. GZ and JH created the mapping population. DR, QM, WC, and SL carried out phenotypic experiments. DR analyzed experimental results. XF and PJ analyzed Illumina 90K assay sequencing data. XM assisted with Illumina sequencing. DR, XW, HW, and LK wrote the manuscript.

## Conflict of Interest Statement

The authors declare that the research was conducted in the absence of any commercial or financial relationships that could be construed as a potential conflict of interest.

## Footnotes

1 http://www.kyazma.nl/

2 http://www.biometris.nl/uk/Software/MapChart/

3 http://en.wikipedia.org/wiki/SPSS

4 http://www.isbreeding.net/

5 https://wheat-urgi.versailles.inra.fr/Seq-Repository/Annotations

## Supplementary Material

FIGURE S1 Overlap of QTLs detected for all of the traits under N^+^ and N^−^ treatment. **(A–E)** Statistical overlap of the QTLs detected for all of the traits under N^+^ and N^−^ conditions are shown by Venn diagrams. The gray circle indicates the QTLs detected only under N^+^ conditions; the white circle indicates QTLs detected only under N^−^ conditions; and the light gray circle indicates the QTLs detected for both conditions.

TABLE S1 Nutrient solution ingredients for wheat seedling growth.

TABLE S2 Phenotype distribution of the parents and individual population.

TABLE S3 ANOVA analysis.

TABLE S4 The integrative genetic map.

TABLE S5 QTL detected for N^+^, N^−^, and G × N.

TABLE S6 Distribution of QTLs detected for growth traits in our study.

TABLE S7 QTL detected for yield traits in mature periods.

TABLE S8 Favorable QTLS/alleles donated by parents in this study.

TABLE S9 Candidate genes.
